# The G protein database, GproteinDb

**DOI:** 10.1093/nar/gkab852

**Published:** 2021-09-27

**Authors:** Gáspár Pándy-Szekeres, Mauricio Esguerra, Alexander S Hauser, Jimmy Caroli, Christian Munk, Steven Pilger, György M Keserű, Albert J Kooistra, David E Gloriam

**Affiliations:** Department of Drug Design and Pharmacology, University of Copenhagen, 2100 Copenhagen, Denmark; Medicinal Chemistry Research Group, Research Centre for Natural Sciences, Budapest H-1117, Hungary; Department of Drug Design and Pharmacology, University of Copenhagen, 2100 Copenhagen, Denmark; Department of Drug Design and Pharmacology, University of Copenhagen, 2100 Copenhagen, Denmark; Department of Drug Design and Pharmacology, University of Copenhagen, 2100 Copenhagen, Denmark; Department of Drug Design and Pharmacology, University of Copenhagen, 2100 Copenhagen, Denmark; Department of Drug Design and Pharmacology, University of Copenhagen, 2100 Copenhagen, Denmark; Medicinal Chemistry Research Group, Research Centre for Natural Sciences, Budapest H-1117, Hungary; Department of Drug Design and Pharmacology, University of Copenhagen, 2100 Copenhagen, Denmark; Department of Drug Design and Pharmacology, University of Copenhagen, 2100 Copenhagen, Denmark

## Abstract

Two-thirds of signaling substances, several sensory stimuli and over one-third of drugs act via receptors coupling to G proteins. Here, we present an online platform for G protein research with reference data and tools for analysis, visualization and design of scientific studies across disciplines and areas. This platform may help translate new pharmacological, structural and genomic data into insights on G protein signaling vital for human physiology and medicine. The G protein database is accessible at https://gproteindb.org.

## INTRODUCTION

Two-thirds of endogenous hormones and neurotransmitters (1), several sensory stimuli and over one-third of the FDA-approved drugs (2) mediate their actions via receptors coupling to G proteins. G proteins are intracellular heterotrimeric proteins consisting of α, β and γ subunits that dissociate to α and βγ upon activation by the G protein-coupled receptor (GPCR). G proteins are named by their α subunit and are divided into four families which share homology and downstream signaling pathways: G_s_ (G_s_ and G_olf_), G_i/o_ (G_i1_, G_i2_, G_i3_, G_o_, G_z_, G_t1_, G_t2_, G_gust_), G_q/11_ (G_q_, G_11_, G_14_ and G_15_) and G_12/13_ (G_12_ and G_13_). The theoretical G protein ‘couplome’ in human spans the potential interaction of these 16 G proteins with ∼800 receptors totaling 12 800 couplings or non-couplings. Recently, breakthroughs in biosensor development (3–6) yielded the first large-scale systematic quantifications of couplings (3,4) which have been unified in a recent meta-analysis (7). The structural elucidation of GPCR–G protein binding currently covers >120 complexes (https://gproteindb.org/structure/gprot_statistics) which make up a majority of the new and nearly all cryo-EM GPCR structures (8). Combined structural and sequence analysis has uncovered GPCR–G protein selectivity determinants (9).

Despite this information, more is needed to realize huge scientific potential. A structural mechanism, ‘conformational selection’ alters G protein selectivity in ligand-dependent ‘signal bias’ (10,11) but we lack the molecular mechanistic understanding to rationally design probes with functional selectivity and drugs with fewer adverse effects. There are no GPCR structure complexes of the G_12/13_ family and receptors with only weak G protein coupling require more insight into their stabilization. The encoding of selectivity in GPCR and G protein sequences has not been described for determinants in the ligand-binding site (12) most important for drug design. Furthermore, G proteins (13) and their signaling interface (14) have recently been identified as (direct) therapeutic targets.

Fully answering these and many related scientific questions would only be possible through a consolidated community data and analysis infrastructure enabling exploitation of the above and coming data in integrative research. Here, we present an extensive online platform for G protein research. The interactive platform features e.g., a G protein coupling atlas, annotated structural templates, interface interactions and matching, and predicted selectivity determinants for mutagenesis. By providing one-stop reference data and accessible data-driven analysis and visualization tools, this platform may help translate more of the many new data into integrative insights and an actionable foundation to advance G protein research across the scientific disciplines and areas.

## MATERIALS AND METHODS

### Coding framework

We built the new resource by re-using the GPCRdb framework (15–17) which uses a Django Framework and the packages BioPython (18), NumPy (19), SciPy (20), and MODELLER (21). For all data browsers (i.e. the couplings, structures, structures models and coupling determinants) we applied the DataTables.js (https://datatables.net) module in conjunction with yadcf.js (https://yadcf-showcase.appspot.com) which support sorting and filtering. The visualizations were written in JavaScript with the largest use of the D3.js framework (https://d3js.org) to generate SVG figures and animations. While initial versions of some resources were published in (9), new data and functionality have been added here along with many new resources tailored for G protein research.

### GPCR–G protein coupling and selectivity resources

**Figure 1.**
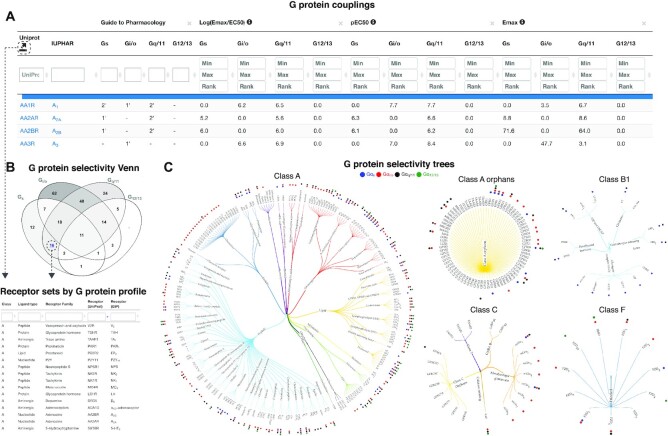
G protein couplings and selectivity. (**A**) G protein couplings encompass Guide to Pharmacology (GtP) primary/secondary transducers (22) and log(*E*_max_/EC_50_), pEC_50_ and *E*_max_ values from profiling studies (3–5) and a means thereof. The confidence and coverage of quantitative couplings can be adjusted based on counts of supporting datasets (default 2) and standard deviations from basal signal (default 1.4). (**B**) G protein selectivity profiles can be intersected in a Venn diagram counting the receptors in each class. (**C**), G protein selectivity trees map couplings to trees classifying receptors by class, ligand type and receptor families sharing endogenous ligand(s). (B, C) Dashed arrows illustrate paths to obtain coupling-based selection of receptor sets for further study.

G protein couplings were filtered, normalized and aggregated onto families as described in (7) and presented in an interactive browser (https://gproteindb.org/signprot/couplings). GPCR–G protein couplings have a confidence filter that by default restricts the quantitative couplings to those supported by a second dataset, while the qualitative (primary versus secondary) couplings in Guide to Pharmacology are instead typically supported by multiple literature references. The requirement for shared coupling is applied on the G protein level—also for families—to avoid the issue of apparent support of family couplings when subtypes differ. G protein coupling selectivity profiles were visualized in a Venn diagram (https://gproteindb.org/signprot/statistics_venn) which, by GPCR class, intersects the receptor sets that couple to the four G protein families. This differs from (9) by adding (i) new datasets ((9) only covered GtP), (ii) a table to select all GPCRs in a class that couple to a given G protein family and (iii) the ability to filter obtained receptor sets based on their classification (lower left in Figure 1). Furthermore, for each GPCR class, we mapped G protein family couplings onto a classification tree of all human receptors by their ligand types (e.g. peptide or aminergic) and receptor families sharing endogenous ligand (https://gproteindb.org/signprot/statistics_tree). This differs from (9) which used (i) a phylogenetic classification (which is arbitrary for many cross-class and orphan receptor comparisons), (ii) GtP data only and (iii) a single cross-class tree (for which receptor names are illegible even in double column figures).

### GPCR–G protein structure models

The GPCR–G protein structure complex models were built by extending the pipeline described in (17). The same steps are used for the homology modelling of the receptor; except, to ensure correct coupling between the receptor and the signaling protein the main template selection is limited to GPCR–G protein complex structures. Only those complex models are built where there is a structure in the GPCR class and the G protein subfamily. The five main template selection criteria are the same for all complex models: (i) GPCR class, (ii) G protein α subunit, or if that is not available: (iii) G protein subfamily, followed by the highest: (iv) GPCR sequence similarity and (v) resolution. The modelling of the G protein α subunit includes the swap-in of an alternative template for the Helical domain when it is missing from the main template; missing loop coordinates get alternative swap-in templates or are freely modeled, and mutated side chains are reverted to wild type.

### GPCR–G protein interface interactions

Pairwise interface interactions were annotated based on geometric rules specified in Supplementary Table S1. Each type of amino acid interaction between a GPCR and a G protein is described on a general and a specific level, where the general level takes the underlying biochemistry into account (e.g. aromatic interaction), while the specific level considers geometric properties (e.g. face-to-edge). Each structural interaction annotation was subsequently enriched with the chemical properties of the participating amino acids. These properties are analyzed to generate an interface interaction fingerprint, a representation of the most conserved properties of the interacting residues across the receptor sequences of all structural templates. This fingerprint can be used to match across all receptors from the same GPCR class to list receptors by decreasing similarity and hence ability to form the same residue and G protein interactions.

### Coupling determinant mutation design

The sequence-based coupling determinants were implemented using our recently published tool to identify sequence signatures (15). Based on the user-specified G protein family and receptor of interest, the tool collects all receptors from the same GPCR class for which coupling data is available. Subsequently, two sets of receptors are created, the ‘couplers’ and the ‘non-couplers’. The sequence signature is calculated as previously described (15) for the two receptor sets spanning all residue positions with an associated generic number. Based on the sequence signature, receptor positions lacking a conserved positive property (score > 10%), which potentially contributes to coupling, are proposed as a mutant by introducing the most conserved amino acid of the binding receptors having that positive property. Vice versa for negative properties (score < −10%), which contribute to non-coupling, mutations are proposed for receptor positions into the most conserved amino acid of the binding receptors lacking that negative property. The inverse approach is applied if the user selects to instead obtain mutations to decrease/abolish coupling to a G protein family. In addition to the suggested mutations, information is provided for each residue position about: sequence conservation, known G protein interactions, known ligand interactions, and the availability of mutation data.

## RESULTS

### GPCR–G protein coupling atlas and selectivity

The *‘G protein couplings’* (https://gproteindb.org/signprot/couplings) integrates data from quantitative profiling studies (3–5) and literature annotation from the Guide to Pharmacology database (22) letting any researcher find and compare couplings in one place (Figure 1A). To ensure confidence and comparability of couplings, all data is consistently filtered and normalized ((7) and Methods). Users can modify coupling confidence or coverage using cut-offs counting the number of supporting datasets or the standard deviations from basal signal. Cross-dataset filtering by mean log(*E*_max_/EC_50_), pEC_50_ and *E*_max_ values can differentiate receptors with strong or no/weak coupling to a G protein subtype or family. Furthermore, a *‘G protein selectivity Venn’* (https://gproteindb.org/signprot/statistics_venn) can discriminate the receptors in each GPCR class by their profile of combined G protein family couplings (Figure 1B). Finally, a *‘G protein selectivity tree’* (https://gproteindb.org/signprot/statistics_tree) maps G protein family couplings onto receptor classification trees – one for each GPCR class and further classified through an alphabetic listing of ligand types (e.g. peptide or lipid) and receptor families sharing endogenous ligands (e.g. serotonin receptors) (Figure 1C). These three resources present complementary means to analyze G protein couplings and to select sets of receptors for further study.

### Structures

**Figure 2.**
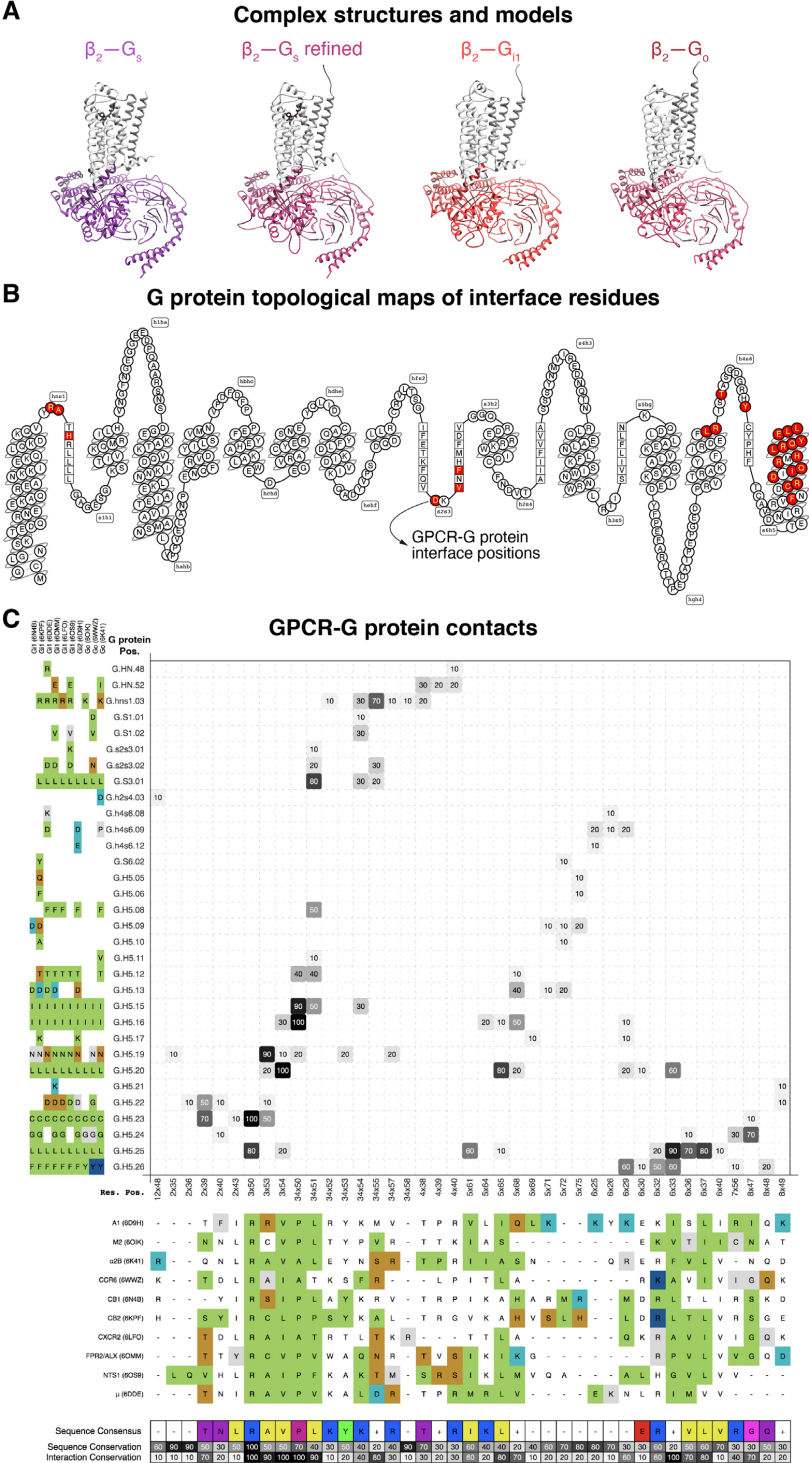
Structures and interface interactions. (**A**) β_2_-adrenoceptor-Gs structure (35), its refined structure and β_2_-G_i1_ and -G_o_ models. (**B**) Snakeplot mapping residues topological segments and highlighting GPCR–G protein interface positions (red residues). (**C**) Interface interactions shown in % and grayscale across class A GPCR–G_i/o_ family structures (interaction presence/absences in each structure is shown upon mouse hover). GPCR and G protein residues are indexed with generic residue numbers (25,26) to compare of structurally corresponding positions and color-coded to visualize properties or interaction types. A structure-based interface fingerprint can be profiled against GPCR lacking structures (Supplementary Figure S2).

The G protein *‘Structures’* (https://gproteindb.org/structure/g_protein_structure_browser) include all unbound and GPCR complex structures from the Protein Data Bank (23). We additionally provide a refined version in which missing or mutated receptor and G_α_ regions are re-modelled based on other more complete structures while mutated residues are reverted to wildtype (Figure 2A). Structure selection is guided by information about G protein family and receptor classification, subunit isoform, species origin, ligand name and modality, structure determination method, resolution and author names (Supplementary Figure S1). The obtained templates can be copied (PDB identifiers) for analysis using GPCRdb's structure comparison tools (24) or exported with their selected data (Excel) for further analysis. Furthermore, this resource is the first to provide *‘Structure models’* (https://gproteindb.org/structure/complex_models) of >3000 GPCR–G protein complexes that are not yet covered by experimental structures but can be modelled based on a similar template from the same GPCR class and G protein family. The structural topology of residue positions can be mapped in a ‘snakeplot’ (Figure 2B) from the *‘G protein page’* (below). The refined and modeled structure complexes expand the GPCR–G protein ‘couplome’ that can be studied across basic and applied structure-based research.

### Receptor–G protein interface

The interfaces of GPCR–G protein structure complexes can be analyzed in the *‘Interface interactions and profiling’* (https://gproteindb.org/signprot/matrix) to identify residue interactions and their frequencies (Figure 2C). Residue interaction frequencies (% and grayscale in Figure 2C) can be analyzed for distribution across structures and filtered to the desired stringency. All residues have generic residue numbers (25,26) for structurally corresponding positions and can be color-coded by properties or interaction types. The property consensus (27) of receptor residues form an interface ‘fingerprint’ which can be matched to a sequence alignment of all GPCRs in the class to profile receptors by their conservation of the interface while inspecting known couplings (Supplementary Figure S2). The platform also features *‘Interface mutations & chimera’* (https://files.gpcrdb.org/GPCR–Gprotein_Mutations.xlsx) from literature annotations (9). These experimental data span modifications of receptors, G proteins or both and their qualitative and quantitate effects. These resources offer unique means for structure-based identification of GPCR–G protein interfaces and for comparison to experimentally characterized GPCR–G protein coupling profiles and determinants.

### Sequence topology, alignment and generic residue numbers

**Figure 3.**
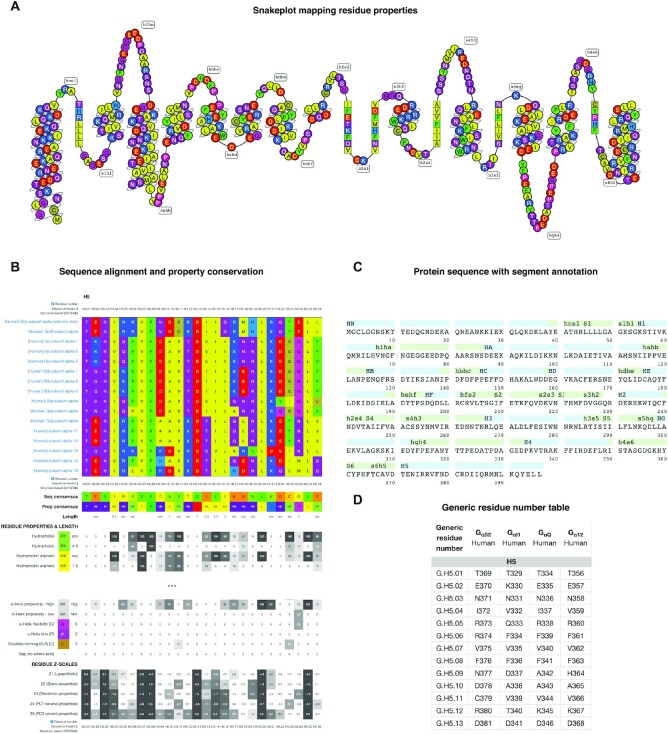
Sequence data in the online G protein research platform. (**A**) Snakeplot visualization of amino acid topological positions and properties. All amino acids are colored by property: polarity for most but backbone modifying ability for proline and glycine and disulphide-formation for cysteine. (**B**) Sequence alignment of the receptor-interacting helix 5 of all human 16 G_α_ proteins along with conservation measures for amino acids and residue groups with similar property and size, and numeric amino acid descriptors, ‘z-scales’ (29) (from https://gproteindb.org/alignment/gproteinselection). (**C**) Protein sequence mapping onto segments by secondary structures (helices, β-sheet and loops). (**D**) Protein-specific and common residue numbers (25) for G_αs_, G_αi1_, G_αq_ and G_α12_ (from https://gproteindb.org/residue/residuetable_gprot). These number tables can also be downloaded in Excel format or retrieved programmatically via a RESTFUL-API web service to integrate the numbering in any dataset and analysis method. (A, B) Taken from the *‘G protein page’* (https://gproteindb.org/signprot) for G_αs_. (A–C), Common residue numbers (25) can be shown by mouse hover.

The *‘G protein page’* (https://gproteindb.org/signprot) summarizes sequence, structural and mutagenesis data. Residue positions are mapped to a ‘snakeplot’ which can be custom colored or display stored functional data about the receptor interface (below), genetic variants (28), post-translational modification sites and a selectivity barcode (9) (Figure 3A). The full-length G_α_ protein sequence mapped to segments by secondary structures (helices, β-sheets and loops) (Figure 3C). The *‘G protein alignments’* (https://gproteindb.org/alignment/gproteinselection) can be customized to cover specific G proteins, sequence segments or common residue number positions (25) (Figure 3B). Each alignment comes with conservation measures for amino acids and residue groups with similar property and size, as well as numeric amino acid descriptors and ‘z-scales’ (29). Furthermore, *‘Generic residue number tables’* (https://gproteindb.org/residue/residuetable_gprot) tabulate G protein-specific and common residue positions (25) (Figure 3D). Together, the sequence alignments, detailed conservation statistics and generic residue numbers provide the foundation for analysis of the sequence basis underlying G protein structure and function, e.g., determinants of molecular interactions and structural conformations.

### Coupling determinant mutation design tool

The *‘Coupling determinant mutation design tool’* (https://gproteindb.org/mutations/gprot_coupling) features data-driven prediction of receptor residue determinants of G protein activation (Supplementary Figure S3). This is based on all couplings (above) and distinct conservation among coupling and non-coupling GPCRs, respectively for a G protein family of interest. The conservation measure goes beyond traditional amino acid identities to analyze groups of residues with similar properties and size allowing shared molecular interactions. Depending on the research question, users can choose to either strengthen or weaken G protein coupling. Accordingly, mutations are suggested to introduce missing and remove conserved consensus amino acids from the receptor set with the desired and undesired coupling status, respectively. Validation is built-in by side-by-side tabulation of suggested mutations with GPCR–G protein interface interactions (above) and effects from literature mutations (15,30), while we invite the research field to feedback new mutagenesis results via a standardized Excel file. Notably, the intersection with interface interactions enables studies focusing on either interface determinants or allosteric modulating G protein binding.

## DISCUSSION

Taken together, the online platform integrates diverse G protein sequence, structure and function data and makes accessible sophisticated analysis tools. The G protein coupling atlas opens for one-stop access to consistently normalized reference data from all major datasets with user-defined confidence and coverage cut-offs tailoring to the needs of each study. For example, functional and mechanistic studies will also benefit from the possibility to distil receptor sets with a specific G protein selectivity based on the atlas and the interactive Venn diagram. Given that there are already over 120 GPCR–G protein complexes (https://gproteindb.org/structure/gprot_statistics) and that these make up the majority of receptor cryo-EM structures, the annotated structures presented herein will help substantially to keep track of new structures and selecting study templates. Furthermore, as cryo-EM structure determination is challenging at a high resolution, the refinement based on other experimental templates model-in many missing sidechains or loop segments. While the vast majority of GPCR–G protein complexes lack an experimental structure, our structure models greatly expand the ‘couplome’ for which we can generate structure-based hypotheses across mutagenesis, dynamics, kinetics, molecular mechanistic and drug design studies. The *‘Interface interactions and profiling’* tool may shed new light on how receptors bind G proteins at the interface (9). However, G protein signaling can also be modulated allosterically (12). Therefore, the *‘Coupling determinant mutation design tool’* presents a unique basis to reveal such allosteric determinants through mutagenesis experiments. Of note, its data differs from the PRECOG server (31) by spanning not one but four datasets (3–5,22)—a necessity to remove unsupported couplings (7)—and its unique residue property-based signatures are integrated with known interface interactions and mutation effects. Hence, we expect that the platform presented here will inspire many studies across basic and applied research and disciplines, aiding the elucidation of, e.g., constitutive activity (32), pre-coupling of G proteins (33,34) and ligand-dependent biased G protein signaling (10).

## DATA AVAILABILITY

All data is available via the web (Section ‘GproteinDb’ in https://gproteindb.org) and GitHub (https://github.com/protwis/gpcrdb_data). Documentation is available at https://docs.gpcrdb.org. All open-source code can be obtained from GitHub (https://github.com/protwis/protwis) under the permissive Apache 2.0 License (https://www.apache.org/licenses/LICENSE-2.0).

## Supplementary Material

gkab852_Supplemental_File

## Notes

Present address: Mauricio Esguerra, Nostrum Biodiscovery, Carrer de Baldiri Reixac 10-12, 08028 Barcelona, Spain.

Present address: Christian Munk, Novozymes A/S, Biologiens Vej 2, 2800 Kongens Lyngby Copenhagen, Denmark.

Present address: Steven Pilger, Explorative Biology and Bioinformatics, LEO Pharma A/S, 2750 Ballerup, Denmark.
