# Supplementary material for: The G protein database, GproteinDb
Source: Nucleic Acids Res. 2021 Sep 27;50(D1):D518–25. doi: 10.1093/nar/gkab852 (PMC8728128; doi:10.1093/nar/gkab852)
Supplement: gkab852_Supplemental_File [file gkab852_supplemental_file.pdf]

# SUPPLEMENTARY DATA

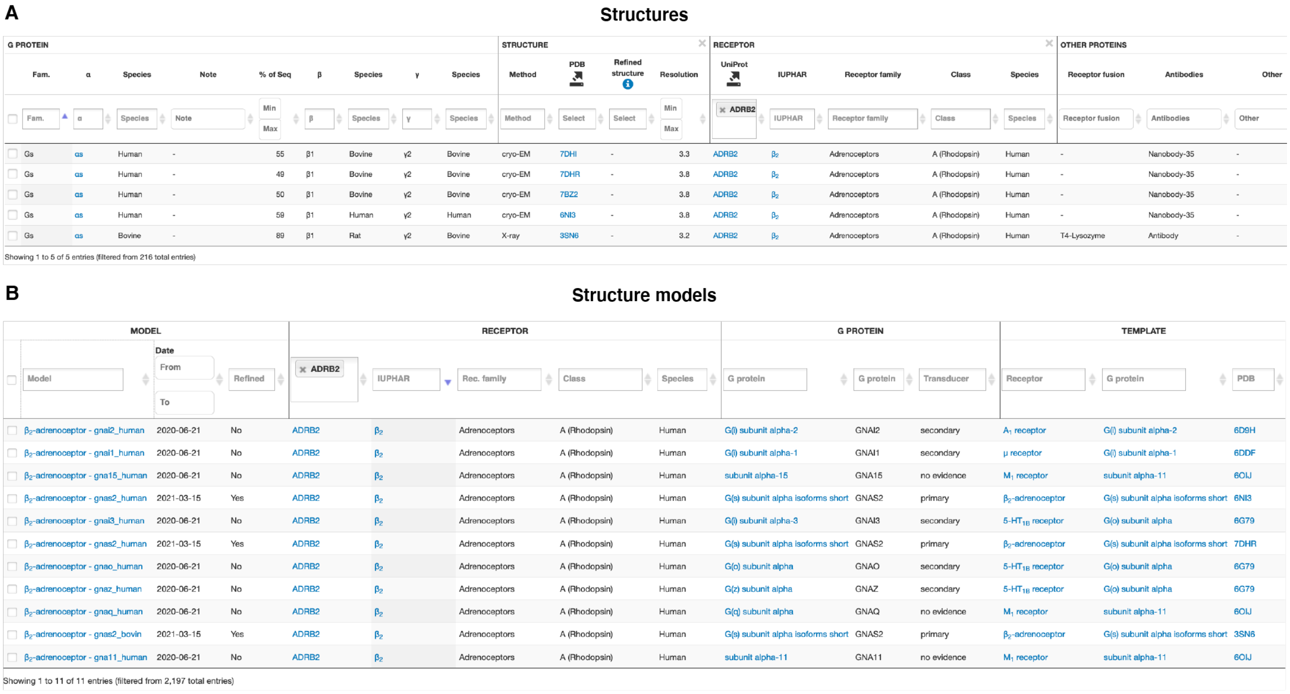

A

Structures

| G PROTEIN | | | | | | | | | STRUCTURE | | | | RECEPTOR | | | | | OTHER PROTEINS | | |
|---|---|---|---|---|---|---|---|---|---|---|---|---|---|---|---|---|---|---|---|---|
| Fam. | α | Species | Note | % of Seq | β | Species | γ | Species | Method | PDB | Refined structure | Resolution | UniProt | IUPHAR | Receptor family | Class | Species | Receptor fusion | Antibodies | Other |
| Gs | αs | Human | - | 55 | β1 | Bovine | γ2 | Bovine | cryo-EM | 7DHI | - | 3.3 | ADRB2 | $\beta_2$ | Adrenoceptors | A (Rhodopsin) | Human | - | Nanobody-35 | - |
| Gs | αs | Human | - | 49 | β1 | Bovine | γ2 | Bovine | cryo-EM | 7DHR | - | 3.8 | ADRB2 | $\beta_2$ | Adrenoceptors | A (Rhodopsin) | Human | - | Nanobody-35 | - |
| Gs | αs | Human | - | 50 | β1 | Bovine | γ2 | Bovine | cryo-EM | 7BZ2 | - | 3.8 | ADRB2 | $\beta_2$ | Adrenoceptors | A (Rhodopsin) | Human | - | Nanobody-35 | - |
| Gs | αs | Human | - | 59 | β1 | Human | γ2 | Human | cryo-EM | 6NI3 | - | 3.8 | ADRB2 | $\beta_2$ | Adrenoceptors | A (Rhodopsin) | Human | - | Nanobody-35 | - |
| Gs | αs | Bovine | - | 89 | β1 | Rat | γ2 | Bovine | X-ray | 3SN6 | - | 3.2 | ADRB2 | $\beta_2$ | Adrenoceptors | A (Rhodopsin) | Human | T4-Lysozyme | Antibody | - |

Showing 1 to 5 of 5 entries (filtered from 216 total entries)

B

Structure models

| MODEL | | | RECEPTOR | | | | | G PROTEIN | | | TEMPLATE | | |
|---|---|---|---|---|---|---|---|---|---|---|---|---|---|
| Model | Date | Refined | ADRB2 | IUPHAR | Rec. family | Class | Species | G protein | G protein | Transducer | Receptor | G protein | PDB |
| $\beta_2$-adrenoceptor - gnai2_human | 2020-06-21 | No | ADRB2 | $\beta_2$ | Adrenoceptors | A (Rhodopsin) | Human | G(i) subunit alpha-2 | GNAI2 | secondary | $A_1$ receptor | G(i) subunit alpha-2 | 6D9H |
| $\beta_2$-adrenoceptor - gnai1_human | 2020-06-21 | No | ADRB2 | $\beta_2$ | Adrenoceptors | A (Rhodopsin) | Human | G(i) subunit alpha-1 | GNAI1 | secondary | μ receptor | G(i) subunit alpha-1 | 6DDF |
| $\beta_2$-adrenoceptor - gna15_human | 2020-06-21 | No | ADRB2 | $\beta_2$ | Adrenoceptors | A (Rhodopsin) | Human | subunit alpha-15 | GNA15 | no evidence | $M_1$ receptor | subunit alpha-11 | 6OIJ |
| $\beta_2$-adrenoceptor - gnas2_human | 2021-03-15 | Yes | ADRB2 | $\beta_2$ | Adrenoceptors | A (Rhodopsin) | Human | G(s) subunit alpha isoforms short | GNAS2 | primary | $\beta_2$-adrenoceptor | G(s) subunit alpha isoforms short | 6NI3 |
| $\beta_2$-adrenoceptor - gnai3_human | 2020-06-21 | No | ADRB2 | $\beta_2$ | Adrenoceptors | A (Rhodopsin) | Human | G(i) subunit alpha-3 | GNAI3 | secondary | 5-$HT_{1B}$ receptor | G(o) subunit alpha | 6G79 |
| $\beta_2$-adrenoceptor - gnas2_human | 2021-03-15 | Yes | ADRB2 | $\beta_2$ | Adrenoceptors | A (Rhodopsin) | Human | G(s) subunit alpha isoforms short | GNAS2 | primary | $\beta_2$-adrenoceptor | G(s) subunit alpha isoforms short | 7DHR |
| $\beta_2$-adrenoceptor - gnao_human | 2020-06-21 | No | ADRB2 | $\beta_2$ | Adrenoceptors | A (Rhodopsin) | Human | G(o) subunit alpha | GNAO | secondary | 5-$HT_{1B}$ receptor | G(o) subunit alpha | 6G79 |
| $\beta_2$-adrenoceptor - gnaz_human | 2020-06-21 | No | ADRB2 | $\beta_2$ | Adrenoceptors | A (Rhodopsin) | Human | G(z) subunit alpha | GNAZ | secondary | 5-$HT_{1B}$ receptor | G(o) subunit alpha | 6G79 |
| $\beta_2$-adrenoceptor - gnaq_human | 2020-06-21 | No | ADRB2 | $\beta_2$ | Adrenoceptors | A (Rhodopsin) | Human | G(q) subunit alpha | GNAQ | no evidence | $M_1$ receptor | subunit alpha-11 | 6OIJ |
| $\beta_2$-adrenoceptor - gnas2_bovin | 2021-03-15 | Yes | ADRB2 | $\beta_2$ | Adrenoceptors | A (Rhodopsin) | Human | G(s) subunit alpha isoforms short | GNAS2 | primary | $\beta_2$-adrenoceptor | G(s) subunit alpha isoforms short | 3SN6 |
| $\beta_2$-adrenoceptor - gna11_human | 2020-06-21 | No | ADRB2 | $\beta_2$ | Adrenoceptors | A (Rhodopsin) | Human | subunit alpha-11 | GNA11 | no evidence | $M_1$ receptor | subunit alpha-11 | 6OIJ |

Showing 1 to 11 of 11 entries (filtered from 2,197 total entries)

**Supplementary Figure 1**. Structures and structure models. (**A**) Structure and (**B**) structure model browsers applying a filter to the receptor UniProt name to show $\beta_2$-G protein complexes.

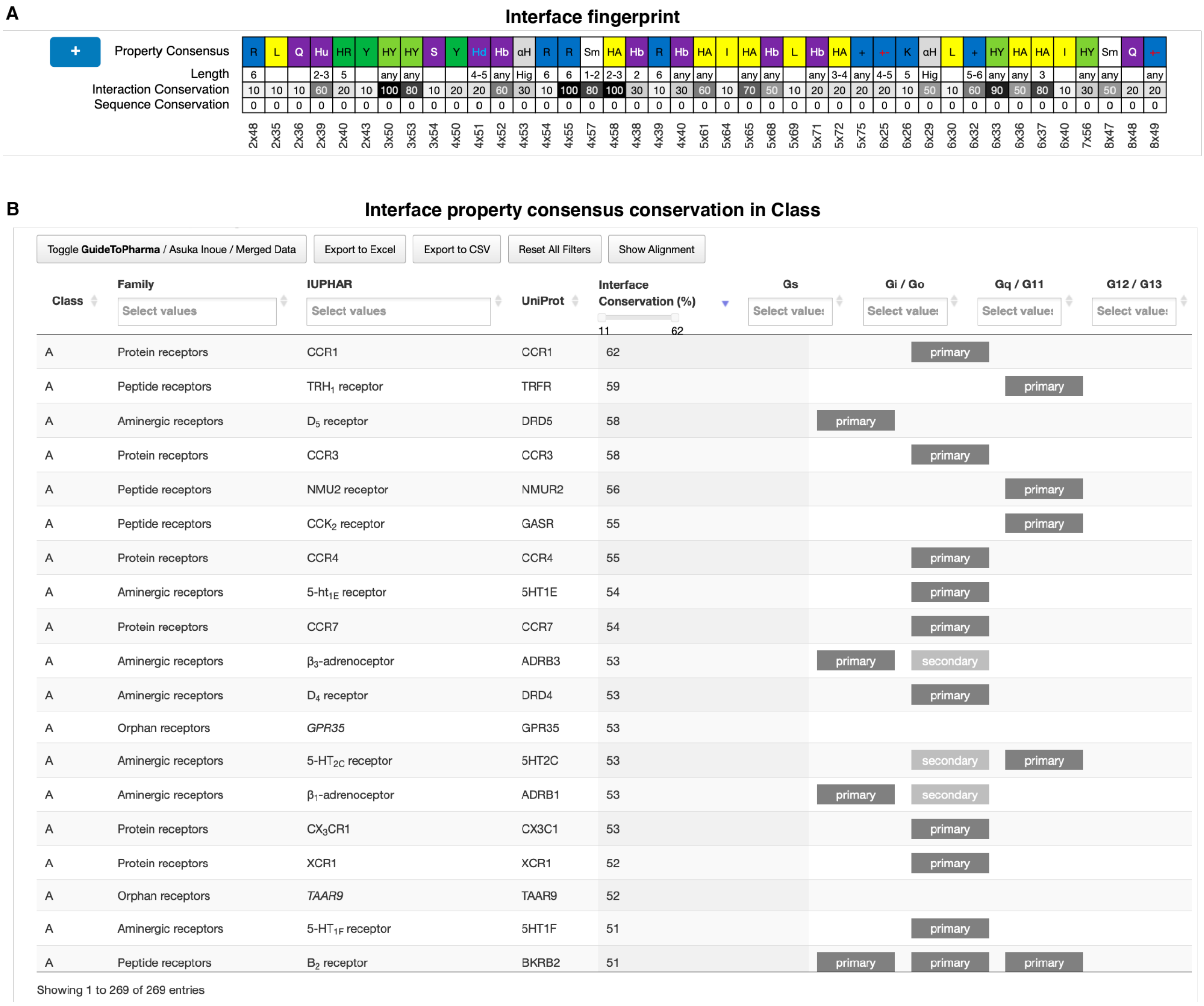


| Class | Family | IUPHAR | UniProt | Interface Conservation (%) | Gs | Gi / Go | Gq / G11 | G12 / G13 |
|---|---|---|---|---|---|---|---|---|
| A | Protein receptors | CCR1 | CCR1 | 62 | | primary | | |
| A | Peptide receptors | $TRH_1$ receptor | TRFR | 59 | | | primary | |
| A | Aminergic receptors | $D_5$ receptor | DRD5 | 58 | primary | | | |
| A | Protein receptors | CCR3 | CCR3 | 58 | | primary | | |
| A | Peptide receptors | NMU2 receptor | NMUR2 | 56 | | | primary | |
| A | Peptide receptors | $CCK_2$ receptor | GASR | 55 | | | primary | |
| A | Protein receptors | CCR4 | CCR4 | 55 | | primary | | |
| A | Aminergic receptors | 5-$ht_{1E}$ receptor | 5HT1E | 54 | | primary | | |
| A | Protein receptors | CCR7 | CCR7 | 54 | | primary | | |
| A | Aminergic receptors | $\beta_3$-adrenoceptor | ADRB3 | 53 | primary | secondary | | |
| A | Aminergic receptors | $D_4$ receptor | DRD4 | 53 | | primary | | |
| A | Orphan receptors | *GPR35* | GPR35 | 53 | | | | |
| A | Aminergic receptors | 5-$HT_{2C}$ receptor | 5HT2C | 53 | | secondary | primary | |
| A | Aminergic receptors | $\beta_1$-adrenoceptor | ADRB1 | 53 | primary | secondary | | |
| A | Protein receptors | $CX_3CR1$ | CX3C1 | 53 | | primary | | |
| A | Protein receptors | XCR1 | XCR1 | 52 | | primary | | |
| A | Orphan receptors | *TAAR9* | TAAR9 | 52 | | | | |
| A | Aminergic receptors | 5-$HT_{1F}$ receptor | 5HT1F | 51 | | primary | | |
| A | Peptide receptors | $B_2$ receptor | BKRB2 | 51 | primary | primary | primary | |

**Supplementary Figure 2.** Interface interactions. (**A**) Interface fingerprint based on residue property consensus of representative class A GPCR-$G_{i/o}$ structures (PDB identifiers: 6OIK, 6D9H, 6K41, 6N4B, 6KPF, 6WWZ, 6LFO, 6OMM, 6OS9 and 6DDE; same as in Fig. 2c). (**B**) Matching of the interface fingerprint listing all other class A GPCRs by decreasing conservation of residue properties along with known G protein couplings.

## Mutations to increase $G_S$ binding

1. Mutations to increase Gs binding | 2. Mutations to decrease Gs binding | 3. All residue positions

**Mutation design:**
To strengthen coupling, mutate receptor positions lacking a conserved positive (+) property by introducing the highest conserved amino acid (AA) of the Gs binding GPCRs having that property.
For negative (-) properties, which contribute to non-binding, instead mutate receptor positions into the highest conserved AA of the Gs binding GPCRs lacking that property.

| Residue | | | | Mutant AA | Structure interactions (# receptors) | | Mutation data ≥5-fold effect | | Gs binders vs non-binders sequence signature | | | | Property conservation Gs binders | | | Property conservation Gs non-binders | | | AA conservation Gs binders | | AA conservation Gs non-binders | | AA conservation GPCR class | |
|---|---|---|---|---|---|---|---|---|---|---|---|---|---|---|---|---|---|---|---|---|---|---|---|---|
| Segment | Target residue number | Generic residue number | Target WT AA | Increase coupling | G protein | Ligand | #Mut. | #Rec. | Score (%) | Property | Length | Matching AAs | Cons. (%) | Property | Length | Cons. (%) | Property | Length | Cons. (%) | AA | Cons. (%) | AA | Cons. (%) | AA |
| TM1 | 43 | 1x42 | I | L | 0 | 4 | 1 | 1 | 31 | αH | Hig | A, K, L, M, R | 98 | HY | any | 90 | HY | any | 38 | L | 35 | V | 31 | I |
| TM3 | 112 | 3x31 | I | F | 0 | 1 | 4 | 2 | -30 | HA | 2-3 | I, L, V | 98 | HY | any | 88 | HY | any | 29 | L | 42 | L | 37 | L |
| TM7 | 312 | 7x38 | N | L | 0 | 44 | 150 | 19 | 27 | HA | 3-4 | I, L, M | 82 | HY | any | 58 | HY | any | 33 | L | 17 | L | 20 | L |
| TM5 | 225 | 5x64 | E | A | 9 | 0 | 2 | 1 | -27 | Hb | any | D, E, H, K,... | 62 | HY | any | 67 | Hb | any | 22 | A | 16 | K | 17 | T |
| TM7 | 320 | 7x47 | G | A | 0 | 0 | 1 | 1 | 25 | HA | any | A, I, L, M, V | 78 | HY | any | 80 | HY | any | 22 | C | 47 | C | 39 | C |
| H8 | 337 | 8x55 | Q | K | 0 | 0 | 0 | 0 | 25 | Hd | 5-6 | K, R, Y | 73 | Hb | any | 59 | Hb | any | 24 | K | 20 | R | 22 | R |
| TM6 | 294 | 6x56 | I | V | 0 | 0 | 2 | 1 | -23 | HA | 3-4 | I, L, M | 91 | HY | any | 89 | HY | any | 20 | I | 39 | L | 36 | L |
| TM4 | 159 | 4x51 | I | A | 0 | 0 | 1 | 1 | 22 | Sm | 1-2 | A, C, S | 91 | HY | any | 84 | HY | any | 31 | A | 32 | V | 23 | V |
| TM5 | 220 | 5x59 | S | V | 0 | 0 | 0 | 0 | -22 | Sm | any | A, C, G, S | 69 | HY | any | 60 | HY | any | 24 | V | 16 | G | 15 | L |
| TM4 | 147 | 4x39 | K | P | 3 | 0 | 0 | 0 | 21 | αH | Min | G, P | 53 | HY | any | 50 | HY | any | 31 | P | 21 | R | 19 | P |
| TM5 | 224 | 5x63 | Q | L | 0 | 0 | 0 | 0 | 21 | L | | L | 69 | αH | Hig | 51 | Hb | any | 31 | L | 26 | R | 26 | R |
| TM7 | 317 | 7x43 | V | A | 0 | 0 | 0 | 0 | 21 | Sm | any | A, C, G, S | 82 | HY | any | 86 | HY | any | 24 | A | 29 | L | 25 | L |
| H8 | 338 | 8x56 | E | L | 0 | 0 | 0 | 0 | 21 | HA | any | A, I, L, M, V | 69 | Hb | any | 86 | Hb | any | 13 | E | 23 | R | 16 | R |
| TM6 | 264 | 6x26 | F | H | 4 | 0 | 0 | 0 | 20 | Ha | 4 | E, H, Q | 60 | Hb | any | 50 | Hb | any | 13 | - | 25 | - | 27 | - |
| H8 | 332 | 8x50 | F | V | 0 | 5 | 1 | 1 | -20 | HY | 4-5 | F, M, Y | 93 | HY | any | 95 | HY | any | 56 | F | 69 | F | 64 | F |
| TM1 | 61 | 1x60 | F | N | 0 | 0 | 0 | 0 | 19 | Hu | 3-4 | H, N, Q | 84 | Hb | any | 81 | Hb | any | 31 | N | 15 | N | 14 | N |
| TM3 | 110 | 3x29 | T | A | 0 | 18 | 77 | 10 | 19 | HA | 1-2 | A, V | 53 | HY | any | 47 | HY | any | 20 | A | 17 | L | 13 | G |
| ICL2 | 139 | 34x51 | F | L | 23 | 0 | 2 | 1 | 19 | L | | L | 87 | HY | any | 78 | HY | any | 49 | L | 30 | L | 31 | L |
| ICL2 | 140 | 34x52 | K | R | 4 | 0 | 0 | 0 | 19 | R | 6 | R | 67 | Hb | any | 66 | Hb | any | 40 | R | 21 | R | 21 | R |
| TM6 | 266 | 6x28 | L | K | 2 | 0 | 0 | 0 | 19 | Hd | 4-5 | H, K, Q | 56 | Hb | any | 54 | Hb | any | 13 | K | 26 | R | 17 | R |

Showing 1 to 20 of 65 entries (filtered from 268 total entries)   Previous 1 2 3 4 Next

CSV Excel

## Mutations to decrease $G_S$ binding

1. Mutations to increase Gs binding | 2. Mutations to decrease Gs binding | 3. All residue positions

**Mutation design:**
To weaken coupling, mutate receptor positions lacking a conserved negative (-) property by introducing the highest conserved amino acid (AA) of the Gs non-binding GPCRs having that property.
For positive (+) properties, which contribute to binding, instead mutate receptor positions into the highest conserved AA of the Gs non-binding GPCRs lacking that property.

| Residue | | | | Mutant AA | Structure interactions (# receptors) | | Mutation data ≥5-fold effect | | Gs binders vs non-binders sequence signature | | | | Property conservation Gs binders | | | Property conservation Gs non-binders | | | AA conservation Gs binders | | AA conservation Gs non-binders | | AA conservation GPCR class | |
|---|---|---|---|---|---|---|---|---|---|---|---|---|---|---|---|---|---|---|---|---|---|---|---|---|
| Segment | Target residue number | Generic residue number | Target WT AA | Decrease coupling | G protein | Ligand | #Mut. | #Rec. | Score (%) | Property | Length | Matching AAs | Cons. (%) | Property | Length | Cons. (%) | Property | Length | Cons. (%) | AA | Cons. (%) | AA | Cons. (%) | AA |
| ICL2 | 141 | 34x53 | Y | A | 11 | 2 | 1 | 1 | 43 | HY | 4-5 | F, M, Y | 82 | HY | any | 62 | Hb | any | 62 | Y | 28 | Y | 35 | Y |
| TM2 | 74 | 2x45 | S | N | 0 | 0 | 0 | 0 | -37 | Hb | 3 | H, N | 91 | Hb | any | 98 | Hb | any | 56 | S | 57 | N | 51 | N |
| TM5 | 223 | 5x62 | F | I | 1 | 1 | 0 | 0 | -37 | HA | any | A, I, L, M, V | 76 | HY | any | 87 | HY | any | 29 | F | 20 | I | 14 | L |
| TM5 | 226 | 5x65 | A | L | 22 | 0 | 10 | 1 | -36 | HA | 2-3 | I, L, V | 89 | HA | any | 93 | HY | any | 42 | A | 54 | L | 46 | L |
| TM1 | 57 | 1x56 | A | V | 0 | 0 | 1 | 1 | 35 | Sm | 0-1 | A, G | 84 | HA | any | 79 | HY | any | 51 | A | 37 | V | 31 | V |
| TM1 | 36 | 1x35 | M | L | 0 | 5 | 4 | 3 | -34 | HA | 2-3 | I, L, V | 67 | HY | any | 83 | HY | any | 16 | L | 25 | L | 27 | L |
| TM5 | 215 | 5x54 | M | I | 0 | 0 | 0 | 0 | -34 | HA | 2-3 | I, L, V | 84 | HA | any | 87 | HA | any | 51 | M | 39 | I | 34 | I |
| ICL2 | 145 | 34x57 | L | R | 6 | 0 | 0 | 0 | 33 | HA | any | A, I, L, M, V | 51 | αH | Hig | 61 | Hb | any | 27 | R | 40 | R | 31 | R |
| TM2 | 73 | 2x44 | T | L | 0 | 0 | 1 | 1 | -33 | αH | Hig | A, K, L, M, R | 80 | HY | any | 81 | HY | any | 38 | V | 36 | L | 33 | L |
| TM4 | 154 | 4x46 | I | C | 0 | 0 | 0 | 0 | 32 | HA | 2-3 | I, L, V | 91 | HY | any | 78 | HY | any | 53 | I | 33 | I | 30 | I |
| TM3 | 107 | 3x26 | E | K | 0 | 1 | 19 | 4 | -32 | + | any | H, K, R | 80 | Hb | any | 85 | Hb | any | 22 | K | 34 | K | 34 | K |
| TM3 | 118 | 3x37 | T | Y | 0 | 15 | 69 | 11 | -32 | HR | 4-5 | F, H, Y | 64 | Hb | any | 67 | Hb | any | 27 | T | 30 | Y | 26 | Y |
| TM4 | 172 | 4x64 | H | F | 0 | 2 | 5 | 2 | -32 | HY | any | A, C, F, I, L... | 42 | - | | 56 | HY | any | 42 | - | 26 | - | 31 | - |
| TM6 | 279 | 6x41 | M | V | 0 | 0 | 2 | 1 | -32 | HA | 1-2 | A, V | 87 | HY | any | 96 | HY | any | 24 | L | 49 | V | 41 | V |
| TM3 | 109 | 3x28 | W | V | 0 | 12 | 57 | 12 | 31 | HR | any | F, H, W, Y | 76 | HY | any | 73 | HY | any | 22 | F | 27 | V | 19 | V |
| TM1 | 47 | 1x46 | I | G | 0 | 1 | 3 | 1 | -31 | G | 0 | G | 62 | Sm | any | 76 | Sm | any | 29 | G | 60 | G | 54 | G |
| TM5 | 229 | 5x68 | Q | R | 22 | 0 | 0 | 0 | 29 | Hd | 4 | H, Q | 76 | Hb | any | 54 | Hb | any | 24 | Q | 22 | R | 17 | R |
| TM6 | 268 | 6x30 | E | K | 2 | 0 | 8 | 4 | 29 | E | 4 | E | 91 | Hb | any | 80 | Hb | any | 51 | E | 22 | E | 25 | E |
| TM2 | 71 | 2x42 | F | Y | 0 | 1 | 0 | 0 | 28 | F | | F | 100 | HY | any | 98 | HY | any | 67 | F | 39 | F | 38 | F |
| TM5 | 218 | 5x57 | V | C | 0 | 1 | 0 | 0 | -28 | C | 2 | C | 87 | HY | any | 86 | HY | any | 20 | A | 44 | C | 38 | C |

Showing 1 to 20 of 131 entries (filtered from 268 total entries)   Previous 1 2 3 4 5 6 7 Next

CSV Excel

**Supplementary Figure 3**. Coupling determinant mutation design. The Coupling determinant mutation design tool ([https://gproteindb.org/mutations/gprot_coupling](https://gproteindb.org/mutations/gprot_coupling)) suggests mutations in a receptor to increase (top) or decrease (bottom) the coupling to a given G protein family (here the β2-adrenoceptor and $G_s$ family). The determinants suggested for mutagenesis are predicted by tailoring the functionality of a sequence signature tool which identifies distinctly conserved residues within one of two sets of receptors (1) (here $G_s$- and non-$G_s$-coupling class A GPCRs).

**Supplementary Table 1.** Distance and angle rules for defining GPCR – G protein interactions. The values are adapted and extended from (2).

| Interaction | Moiety 1 | Moiety 2 | Max distance (Å) | Angle rule |
|---|---|---|---|---|
| Hydrogen bond (strong) | Donor | Acceptor | 3.5 | $\langle \overrightarrow{\text{Donor} - \text{Hydrogen}}, \overrightarrow{\text{Hydrogen} - \text{Acceptor}} \rangle \in \left[\frac{-\pi}{3}, \frac{\pi}{3}\right]$ |
| Hydrogen bond (weak) | Donor | Acceptor | 4.0 | |
| Ionic | Cation | Anion | 4.0 | |
| Hydrophobe | Hydrophobic atom | Hydrophobic atom | 4.5 | |
| Aromatic (Face to face) | Ring center | Ring center | 4.4 | $\langle \overrightarrow{ring\ normal_1}, \overrightarrow{\text{ring}\ normal_2} \rangle \in [\frac{-\pi}{6}, \frac{\pi}{6}]$ |
| Aromatic (Edge to face) | Ring center | Ring center | 5.5 | $\langle \overrightarrow{ring\ normal_1}, \overrightarrow{\text{ring}\ normal_2} \rangle \in [\frac{\pi}{6}, \frac{5\pi}{6}]$ |
| Aromatic ($\pi$-cation) | Ring center | Cation | 6.6 | $\langle \overrightarrow{ring\ normal}, \overrightarrow{\text{ring center} +} \rangle \in [\frac{-\pi}{6}, \frac{\pi}{6}]$ |
| Van-der-Waals (vdW) | vdW Atom | vdW Atom | 1.1*atom radii sum | |

## REFERENCES

1. Kooistra, A.J., Mordalski, S., Pándy-Szekeres, G., Esguerra, M., Mamyrbekov, A., Munk, C., Keserű, G.M. and Gloriam, David E. (2021) GPCRdb in 2021: integrating GPCR sequence, structure and function. *Nucleic Acids Res.*, **49**, D335-D343.

2. Marcou, G. and Rognan, D. (2007) Optimizing fragment and scaffold docking by use of molecular interaction fingerprints. *J. Chem. Inf. Model.*, **47**, 195-207.
